# Outcomes of a Multidisciplinary Team in the Management of Patients with Early-Stage Breast Cancer Undergoing Neoadjuvant Chemotherapy at a Community Cancer Center

**DOI:** 10.3390/curroncol30050366

**Published:** 2023-05-08

**Authors:** Prarthna V. Bhardwaj, Holly Mason, Seth A. Kaufman, Paul Visintainer, Grace Makari-Judson

**Affiliations:** 1 Division of Hematology—Oncology, University of Massachusetts Chan Medical School—Baystate, 759 Chestnut Street, Springfield, MA 01199 , USA; 2 Breast Surgery Section, University of Massachusetts Chan Medical School—Baystate, 759 Chestnut Street, Springfield, MA 01199, USA; 3 Division of Radiation Oncology, University of Massachusetts Chan Medical School—Baystate, 759 Chestnut Street, Springfield, MA 01199, USA; 4 Institute for Healthcare Delivery and Population Science, University of Massachusetts Chan Medical—Baystate, 759 Chestnut Street, Springfield, MA 01199, USA

**Keywords:** neoadjuvant chemotherapy, breast cancer, multidisciplinary team, care pathway, breast cancer therapy, breast surgery, breast radiation therapy

## Abstract

*Background:* The utilization of neoadjuvant chemotherapy (NAC) remains highly variable in clinical practice. The implementation of NAC requires coordination of handoffs between a multidisciplinary team (MDT). This study aims to assess the outcomes of an MDT in the management of early-stage breast cancer patients undergoing neoadjuvant chemotherapy at a community cancer center. *Methods:* We conducted a retrospective case series on patients receiving NAC for early-stage operable or locally advanced breast cancer coordinated by an MDT. Outcomes of interest included the rate of downstaging of cancer in the breast and axilla, time from biopsy to NAC, time from completion of NAC to surgery, and time from surgery to radiation therapy (RT). *Results:* Ninety-four patients underwent NAC; 84% were White and mean age was 56.5 yrs. Of them, 87 (92.5%) had clinical stage II or III cancer, and 43 (45.8%) had positive lymph nodes. Thirty-nine patients (42.9%) were triple negative, 28 (30.8%) were human epidermal growth factor receptor (HER-2)+, and 24 (26.2%) were estrogen receptor (ER) +HER-2−. Of 91 patients, 23 (25.3%) achieved pCR; 84 patients (91.4%) had downstaging of the breast tumor, and 30 (33%) had axillary downstaging. The median time from diagnosis to NAC was 37.5 days, the time from completion of NAC to surgery was 29 days, and the time from surgery to RT was 49.5 days. *Conclusions:* Our MDT provided timely, coordinated, and consistent care for patients with early-stage breast cancer undergoing NAC as evidenced by time to treatment outcomes consistent with recommended national trends.

## 1. Introduction

Modern breast cancer management has become increasingly complex and specialized over the years. A multidisciplinary approach to cancer care that brings together all pertinent disciplines to discuss optimal care is not only attractive but also promoted in cancer care guidelines [1]. Neoadjuvant chemotherapy (NAC) in breast cancer has historically been reserved for patients with large, inoperable tumors or inflammatory breast cancer, but is now being considered for women with operable disease as well. Larger clinical trials such as EORTC 10902 and NSABP B-18 have shown no differences between the same systemic therapy given pre- or post-surgery on disease-free survival (DFS) and overall survival (OS) [2,3,4]. However, the purpose of administering chemotherapy prior to surgery is to downstage the tumor and provide information regarding treatment response. Downstaging the tumor may allow less extensive surgery on the breast and axilla, enabling patients to undergo breast conservation surgery instead of mastectomy, improve cosmetic outcomes, and reduce postoperative complications such as lymphedema [5,6]. Several randomized trials have shown that the frequency of mastectomies was decreased using NAC as opposed to adjuvant systemic treatment [2,7].

NAC can also eliminate axillary nodal metastases [7]. While sentinel lymph node biopsy (SLNB) is widely accepted post-NAC for patients who are clinically node-negative at presentation [8], the management of the axilla in patients who present with nodal metastases and appear to downstage with NAC remains controversial. Mamtani et al. determined the ability to avoid axillary lymph node dissections at the time of surgery in nearly 50% of patients with node-positive disease after receiving NAC [6]. 

NAC is also now being used to tailor adjuvant therapies for patients with human epidermal growth factor receptor (HER-2) positive and triple-negative breast cancers (TNBC) based on the presence or absence of minimal residual invasive disease in the breast or lymph nodes [9,10]. Early response after two to three cycles of NAC is thought to be a predictor of pathologic complete response (pCR) and may therefore serve as a predictor for long-term outcome [11]. Studies have also shown that the rate of pCR in patients with TNBC receiving NAC is significantly higher than that of non-TNBC patients [12,13].

Although there is common consensus on the patient subgroups most likely to benefit from NAC in breast cancer [14,15], its utilization in clinical practice remains highly variable. Candidacy for receiving NAC is carefully determined based on discussions between breast surgeons, medical oncologists, radiation oncologists, pathologists, and radiologists. Optimized care of breast cancer patients undergoing NAC requires coordination within the multidisciplinary care team (MDT) to streamline care through multiple handoffs between specialties to minimize unnecessary delays and provide consistent, continuous, coordinated, and improved care to patients with early-stage breast cancer. MDT and the collegial discussion of patient cases offer the benefits of an optimal approach to therapy in a simple and practical way. In most cases, patients feel more comfortable knowing that their situation has been evaluated and discussed by different health care professionals and the teams caring for them are communicating effectively.

While most of the data regarding patterns of NAC use in early-stage operable breast cancer are available from larger clinical trials and academic institutions, there is a paucity of real-life data describing the contemporary use of NAC in community cancer centers and the feasibility as well as outcomes of the MDT. Our study aims to evaluate the process of this MDT at our institution in the management of early-stage breast cancer patients undergoing neoadjuvant chemotherapy.

## 2. Methods

This was a retrospective case-series conducted at Baystate Medical Center, a 715-bed academic teaching hospital in Western Massachusetts. We included patients seen at our cancer center between October 2018 and October 2020. All patients diagnosed with early-stage operable and locally advanced breast cancer who have undergone NAC with intent for surgical resection post-treatment at our institution were included in this study. Patients with metastatic breast cancer at the time of diagnosis were excluded. Patients who underwent surgery or radiation therapy at a different facility were also excluded. 

### 2.1. Outcomes

Outcomes included the proportion of pathologic complete response, proportion of downstaging of cancer in the breast, proportion of downstaging in the axilla, proportion of clinical trial enrollment, quality measures including timeliness of referral back to the breast surgeon during NAC, referral back to radiation oncologist, time from biopsy to NAC, time from completion of NAC to surgery, and time from surgery to radiation therapy (RT). Evaluation of our MDT was based on our quality measures or time to treatment outcomes in comparison with national standards, which is the focus of our study.

### 2.2. Data Collection

The total number of patients diagnosed with Stage I–III breast cancer during the study period presenting to our cancer center was obtained from our breast cancer tumor registry, which tracks all our early-stage breast cancer patients. The patients receiving NAC were obtained from our NAC registry maintained by a breast cancer intake coordinator, a unique list in our password-protected electronic health record (EHR) established for internal quality improvement purposes only.

Patient and tumor characteristics, management aspects, and outcomes measures were obtained from the EHR, Cerner-powered CIS at our institution. These data were entered into Research Electronic Data Capture (REDCap) [16]. A single author entering all the pertinent patient data ensured uniformity in data collection.

For pCR to be designated in this study, there must have been no histologic evidence of invasive cancer, either in the breast or axillary lymph nodes following definitive surgery. The presence of ductal carcinoma in-situ (DCIS) was disregarded, given that this was not thought to affect the systemic risk of recurrence [17]. 

We defined downstaging as decreasing the size, extent of metastases, and/or lymph node involvement of a tumor using anti-cancer therapy. 

### 2.3. Analysis

As a case series, data analyses were limited to descriptive statistics. No hypothesis testing was conducted. We utilized descriptive statistics, including means, median, and standard deviations (SD) for continuous variables and counts and proportions for categorical variables to summarize patients’ demographic and clinical characteristics. 

### 2.4. MDT

**Figure 1:**
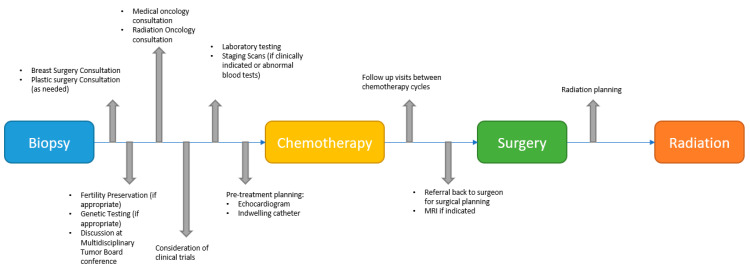
Clinical pathway involving multidisciplinary team-driven care for the management of patients undergoing neoadjuvant chemotherapy for early-stage breast cancer.

To place MDT in context, we have summarized our conceptualization and process of modern MDT-driven care as available at our cancer center in Figure 1. Baystate Health Breast Network involves breast surgeons, medical oncologists, radiation oncologists, pathologists and radiologists who meet quarterly and are responsible for creating guidelines to standardize various breast cancer related practices across the institution. Through this endeavor, guidelines have been created for candidacy for neoadjuvant systemic therapy as described in Supplementary Table S1. All patients who undergo a breast biopsy at our institution are automatically referred to a breast surgeon, who will then determine the timing of referral to a medical oncologist based on their candidacy for neoadjuvant therapy versus upfront surgery. All potential neoadjuvant therapy candidates based on available guidelines are presented at our weekly virtual tumor board conference for a team consensus on best approach to treatment. Once it has been determined that a patient will initiate NAC, they are referred to medical oncology. A breast cancer clinical coordinator oversees the care process during the pre-operative period to ensure that patients are appropriately referred for their labs and scans, and also referred back to the surgeons more than midway through NAC to avoid delays in surgical planning. All patients who are referred to medical oncology are initially referred to radiation oncology as well. Patients are also provided with a handout with all the steps and appointments delineated in their handout at the time of their initial medical oncology visit. Samples of this handout are available in the Supplement.

## 3. Results

A total of 54 patients were eventually diagnosed with TNBC stage II, or III between October 2018 and October 2020. Of these patients, 39 (68.4%) were referred to receive NAC. Forty patients were diagnosed with HER-2 positive breast cancer stage II, or III during the study period, of which 28 (66.6%) received NAC. Seventy-eight patients were diagnosed with ER/PR positive HER-2 negative breast cancer stage II or III, of which 24 (30.7%) underwent NAC. This study did not assess the number of patients who may have met the criteria for NAC and were not referred for NAC.

### 3.1. Patient and Tumor Characteristics

A total of 94 patients underwent NAC. Of these, 84% were White, 12.8% were Black and 3.2% were Asian. This demographic was reflective of all patients presenting to our cancer center with a new diagnosis of breast cancer as available from our breast cancer registry. The mean age was 56.5 years. Of these patients, 87 (92.5%) had clinical stage II or III cancer, and 43 (45.8%) had positive lymph nodes. Thirty-nine patients (42.9%) were triple negative, 18 (19.8%) were ER positive and HER-2 positive, 10 (11.0%) were ER negative and HER-2 positive and 24 (26.2%) were ER positive and HER-2 negative. The most common indications for NAC were to downstage the axilla (42.6%) and for HER-2 tailoring of treatment (25.5%). Several patients had one or more of these indications, as described in Table 1.

### 3.2. Pathological Complete Response

Of the 91 patients who underwent NAC with complete data, 23 (25.3%) achieved a pathologic complete response (pCR). Of these 23 patients, 12 (52.2%) had ER-negative, HER-2-low or negative cancer, 7 (30.4%) had ER-negative HER-2-positive cancer, 3 (13.0%) had ER-positive HER-2-positive cancer, and 1 (4%) had ER-positive HER-2-negative cancer (Table 2).

### 3.3. Other Outcomes

The median time from diagnosis of breast cancer to initiation of NAC was 37.5 days (ranging between 3 and 150 days). Eighty-five (91.4%) patients had downstaging of their breast tumor, and 31 (33%) had axillary downstaging with 53 (57.6%) patients undergoing a lumpectomy while 39 (41.9%) underwent a mastectomy and 22 (23.7%) patients went on to have bilateral mastectomy. A third of patients (33%) had downstaging of axilla based on final surgical pathology (Supplementary Table S2). 

**Figure 2:**
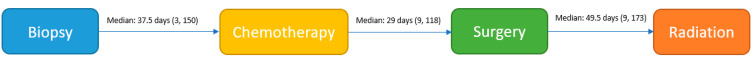
Time to treatment components including time from diagnosis to initiation of neoadjuvant chemotherapy (NAC), time from completion of NAC to surgery, time from surgery to radiation therapy. One patient did not follow through with appointments due to personal conflicts, reflecting the ranges in the median.

All patients followed back with their surgeons before completion of NAC. The median time from completion of NAC to definitive surgery was 29 days (ranging between 9 to 118 days). Of the 78 patients who received adjuvant radiation, all had a radiation oncology consultation before surgery. However, 48 (51.1%) patients returned to see their radiation oncologist before completion of NAC, of which 67.4% were lymph node positive. The median duration of radiation therapy was 33 days (ranging between 12 to 73 days). Five patients (6.4%) underwent radiation therapy for more than two weeks beyond the expected time of completion (i.e., 4–6 weeks based on standard vs. hypo-fractionated RT). The mean time from surgery to radiation therapy was 49.5 days (ranging from 9 to 173 days) (Table 3). Time to treatment outcomes in the context of our MDT have been illustrated in Figure 2.

## 4. Discussion

In our study assessing the outcomes of an MDT in managing breast cancer, the median time from completion of NAC to surgery was less than a month. Various studies have established superior overall survival and 5-year recurrence-free survival in patients undergoing surgery within 8 weeks of completion of NAC. There has been a suggested increase in RCB class and a decline in pCR rates after a 4-week interval between chemotherapy and surgery, and worse overall survival after an 8-week interval [18,19,20,21]. This observation reinforces the importance of referring patients back to their surgeons in a timely fashion for surgical planning which was noted in our study. All patients saw radiation oncology at least once pre-operatively. Although not all patients were referred back to the radiation oncologist prior to completion of NAC, the mean time from surgery to initiation of radiation therapy was 7 weeks. It is worthy of note that a few patients required second surgeries, including re-excision of margins or complete axillary dissection based on pathology results that delayed the initiation of radiation therapy. Despite this, we were aligned with providing radiation therapy at an optimal recommended interval of within 8 weeks after surgery, which has been associated with better disease-free survival and overall survival [22,23]. The median time from diagnosis of cancer to initiation of NAC was less than 6 weeks. Time to treatment initiation is considered an important metric from a patient perspective, as delays provoke anxiety and are thought to influence long-term outcomes. This perception of longer wait times equating to poorer outcomes may be magnified by the role of mammograms whose prerogative is ‘early detection saves lives’; conversely, delays are perceived to result in mortality. Various factors influence the time to start of NAC including additional testing, for e.g., MRI, staging studies, and fertility preservation as indicated. Prior studies have demonstrated no impact on long term patient outcomes so long as NAC is initiated within 8 weeks of diagnosis [24,25,26]. 

Overall pCR rates in our patients were noted to be lower than those demonstrated by larger clinical trials however similar or improved compared to other real-world studies [27,28,29]. Of the patients who achieved pCR, the majority were ER-negative and HER-2-negative, followed by HER-2-positive patients irrespective of ER status. Traditionally, pCR rates are highest in HER-2-positive patients [17]. pCR rates are likely influenced by multiple factors and the small sample size. While assessing treatment regimens used for patients with HER-2 positive disease in our study, a few patients did not receive dual HER-2-based therapies in the neoadjuvant setting. We hypothesize that variable physician prescribing trends during the study period could attribute to lower pCR rates in HER-2-positive patients and hence, the overall population. Despite lack of pCR, most of the patients had at least partial response in the breast and a third had axillary downstaging, resulting in a more conservative axillary approach surgically. Axillary pCR rates remain variable and are affected by age, molecular subtype, tumor grade and Ki-67 [30,31,32].

Coordinated care through an MDT has previously shown to improve receipt of treatment, adherence to treatment recommendations and overall survival, including in vulnerable cancer populations being treated at safety net hospitals [33,34,35]. It can level the playing field for patients from various socioeconomic backgrounds and thus, serve as a bridge to overcome disparities in access to care. 

Our study had key limitations which include a smaller sample size, given this is a single-institution study. This study did not specifically evaluate how many patients were appropriately referred for neoadjuvant therapies as it was assumed that patients were appropriately referred based on institutional guidelines as referenced in the Supplementary Materials. We did not collect data regarding omission of treatments in subsequent cycles or interruptions in chemotherapy cycles due to various factors, including age, co-morbidities and adverse effects which could have resulted in fewer cycles than intended, resulting in lower overall pCR rates. However, this study can serve as a model for how an MDT can be utilized in ensuring adherence to quality metrics, which can in turn improve long-term patient outcomes.

Although our small sample size did not allow for examining differences in patient subsets, using our standardized clinical pathway model for every new patient with a diagnosis of breast cancer requiring NAC allows high standards for all patients irrespective of race or ethnicity.

## 5. Conclusions

In our study, the multidisciplinary care process resulted in timely, coordinated, and consistent care for all patients with early-stage breast cancer undergoing NAC. All patients were referred back to the surgeon prior to completion of NAC for surgical planning. The median time to treatment initiation, time from completion of NAC to surgery and time from surgery to radiation were within recommended intervals for optimal long-term patient outcomes. NAC will likely be used in an increasing fashion as the indications expand, especially in smaller cancers that are triple negative and HER-2 positive. Hence, there is a need not only to advance systemic therapies, but also to create a streamlined process to optimize outcomes. To that effect, our multidisciplinary care pathway as described can serve as a model for growing community cancer centers to address disparities in care.

## Figures and Tables

**Table 1:** Patient characteristics.

Patient Characteristics	Overall	Pathological Complete Response
N (%)	94 (100)	23 (24.5)
Age (mean)	56.5 (12.8)	54.4 (12.1)
Race		
White	79 (84.0)	19 (82.6)
Black	12 (12.8)	2 (8.7)
Asian	3 (3.2)	2 (8.7)
Ethnicity		
Hispanic	11 (11.7)	1 (4.3)
Non-Hispanic	82 (88.3)	21 (95.7)
ECOG Performance Status		
0	78 (83.0)	20 (87.0)
1	12 (12.8)	3 (13.0)
2	1 (1.1)	0 (0.0)
Not documented	3 (3.2)	0 (0.0)
Prior Breast Cancer (DCIS or invasive)	11 (12.0)	2 (8.7)
Clinical Stage		
I	5 (5.3)	0 (0.0)
II	66 (70.2)	15 (65.2)
III	21 (22.3)	8 (34.8)
Clinical Tumor Stage		
TI	10 (10.6)	1 (4.3)
T2	60 (63.8)	12 (52.2)
T3	19 (20.2)	9 (39.1)
T4	3 (3.2)	1 (4.3)
Tx	2 (2.1)	0 (0.0)
Clinical Lymph Node Stage		
N0	51 (54.3)	12 (52.1)
N1	38 (40.4)	9 (39.1)
N2	4 (4.3)	1 (4.3)
N3	1 (1.1)	1 (4.3)
ER Receptor Status		
Positive	44 (46.8)	4 (17.4)
Negative	50 (53.2)	19 (82.6)
PR Receptor Status		
Positive	36 (38.3)	2 (8.7)
Negative	58 (61.7)	19 (82.6)
HER-2 Neu Receptor Status		
Positive	28 (29.8)	10 (43.5)
Negative	66 (70.2)	13 (56.5)
Chemotherapy Regimen		
DDAC/T	32 (34.0)	4 (17.4)
DDAC/TC	15 (16.0)	6 (26.1)
TC	9 (9.6)	0 (0.0)
TCHP	22 (23.4)	10 (43.5)
THP	3 (3.2)	1 (4.3)
Time from diagnosis (1st breast biopsy) to NAC (in days)—median (min, max)	37.5 (3, 150) *	41.0 (21, 98)
Indication for NAC		
Less Extensive Surgery	6 (6.4)	3 (13.0)
HER2 tailoring of treatment	24 (25.5)	9 (39.1)
Inoperable to Operable	12 (12.8)	2 (8.7)
Operable Mastectomy to BCS	12 (12.8)	1 (4.3)
Time for genetics	21 (22.3)	6 (26.1)
Time for Surgical Planning	12 (12.8)	3 (13.0)
Lymph Node positive to negative	40 (42.6)	12 (52.2)
Time from completion of NAC to surgery (in days)—median (min, max)	29.0 (9, 118) ^*^	30.0 (13, 48)

Abbreviations: DCIS: Ductal Carcinoma In Situ, DDAC/T: Dose-dense doxorubicin and cyclophosphamide to paclitaxel, DDAC/TC: Dose-dense doxorubicin and cyclophosphamide to paclitaxel and carboplatin, TC: docetaxel and cyclophosphamide, TCHP: docetaxel, carboplatin, trastuzumab and pertuzumab, THP: paclitaxel, trastuzumab and pertuzumab, NAC: Neoadjuvant chemotherapy, ER: Estrogen receptor, HER2: Human Epidermal Growth Factor Receptor 2. * One patient did not follow through with the treatment plan regularly, resulting in delays in treatment.

**Table 2:** Pathological Complete Response by Tumor Type.

Tumor Type	Total	RCB 0 [pCR]	RCB I	RCB II	RCB III
N (%)	91 (100.0)	23 (25.3)	19 (20.9)	38 (41.8)	11 (12.1)
ER + HER2 +	18 (19.8)	3 (16.7)	1 (5.6)	12 (66.7)	2 (11.1)
ER + HER2 −	24 (26.4)	1 (4.2)	2 (8.3)	14 (58.3)	7 (29.2)
ER-HER2 +	10 (11.0)	7 (70.0)	3 (30.0)	0 (0.0)	0 (0.0)
ER-HER2 −	39 (42.9)	12 (30.8)	13 (33.3)	12 (30.8)	2 (51.3)

Abbreviations: ER: Estrogen receptor, HER2: Human Epidermal Growth Factor Receptor 2, RCB: Residual Cancer Burden, pCR: Pathologic complete response.

**Table 3:** Quality Metrics to assess outcomes of multidisciplinary teams.

	Overall	Clinical Node Positive	Clinical Node Negative
N (%)	94 (100.0)	43 (45.7)	51 (54.3)
Follow up with surgeon prior to completion of NAC—Yes	92 (98.9)	42 (100.0)	50 (98.0)
Follow up with radiation oncology prior to completion of NAC—Yes	48 (51.1)	29 (67.4)	19 (37.3)
Enrollment in clinical trial—Yes	5 (21.7)	3 (21.4)	2 (22.2)
Time from surgery to RT (in days)—median (min, max)	49.5 (9, 173) N = 78	55 (9, 173) N = 43	48 (25, 140) N = 35
Time to complete RT (in days)—median (min, max)	33.0 (12, 73)	39.0 (12, 73)	29.0 (21, 52)
Duration of RT for more than 2 weeks beyond expected time—Yes	5 (6.4)	4 (9.3)	1 (2.9)

Abbreviations: NAC: Neoadjuvant chemotherapy, RT: Radiation therapy. One patient did not follow through with the treatment plan regularly, resulting in delays in treatment.

## Data Availability

The data presented in this study are available on request from the corresponding author.

## Supplementary Materials

The following supporting information can be downloaded at: https://www.mdpi.com/article/10.3390/curroncol30050366/s1, Table S1: Indications for consideration of neoadjuvant systemic therapy; Table S2: Management after neoadjuvant chemotherapy.
