# Supplementary material for: Outcomes of a Multidisciplinary Team in the Management of Patients with Early-Stage Breast Cancer Undergoing Neoadjuvant Chemotherapy at a Community Cancer Center
Source: Curr Oncol. 2023 May 8;30(5):4861–70. doi: 10.3390/curroncol30050366 (PMC10217230; doi:10.3390/curroncol30050366)
Supplement: Supplementary file 1 [file curroncol-30-00366-s001.zip › Pre-op Chemo Locally Advanced Breast Cancer.pdf]

BAYSTATE HEALTH BREAST NETWORK

Pre-OP Chemotherapy Clinical Pathway

Locally Advanced Breast Cancer

Non-Operable IIIA, IIIB, IIIC

DATE	PHYSICIAN VISIT	TEST DATE	EVALUATION	TREATMENT
	BREAST SURGEON CONSULTATION		Core Biopsy	
			Breast Imaging	
	MEDICAL ONCOLOGIST CONSULTATION			
			Pre-Chemo Labs	
			PET/CT, +/- Bone Scan<br>+/- or CT Chest + Abdomen + pelvis	
			Pretreatment Planning<br>Cardiac ECHO<br>Port-a-cath	
			GENETIC TESTING<br>if appropriate	
			FERTILITY PRESERVATION<br>if appropriate	REFERRAL
	RADIATION ONCOLOGY CONSULTATION			
	MEDICAL ONCOLOGIST		TREATMENT PLAN:<br>Teaching Visit<br>Follow-up visits between Chemotherapy Cycles	CHEMOTHERAPY:<br>Regimen:<br>Number of Weeks:<br><br>TARGETED TREATMENT:<br>Regimen:<br>Number of Weeks
After cycle #6/8 or #4/6	BREAST SURGEON		Surgical Planning Visit	
	BREAST SURGEON			SURGERY<br>+/- Port Removal
2-3 wks. post-op	RADIATION ONCOLOGIST		Radiation Planning	RADIATION
2-3 wks. post-op	MEDICAL ONCOLOGIST		Discussion of additional treatments<br>If indicated	Hormone treatment<br>Targeted treatment<br>Clinical Trials
