# Supplementary material for: Outcomes of a Multidisciplinary Team in the Management of Patients with Early-Stage Breast Cancer Undergoing Neoadjuvant Chemotherapy at a Community Cancer Center
Source: Curr Oncol. 2023 May 8;30(5):4861–70. doi: 10.3390/curroncol30050366 (PMC10217230; doi:10.3390/curroncol30050366)
Supplement: Supplementary file 1 [file curroncol-30-00366-s001.zip › Pre-OP Chemo Operable Stage IIA-IIB-IIIA.pdf]

BAYSTATE HEALTH BREAST NETWORK

Pre-OP Chemotherapy Clinical Pathway

Operable Stage IIA, IIB, IIIA*

DATE	PHYSICIAN VISIT	TEST DATE	EVALUATION	TREATMENT
	BREAST SURGEON CONSULTATION		Core Biopsy	
			Breast Imaging	
	MEDICAL ONCOLOGIST CONSULTATION		Blood tests	
			(Scans if clinically indicated or abnormal blood tests)	
			Pretreatment Planning<br>Cardiac ECHO<br>Port-a-Cath	
			FERTILITY PRESERVATION if appropriate	REFERRAL
			GENETIC TESTING if appropriate	
	RADIATION ONCOLOGIST CONSULTATION		Pre Treatment evaluation and Consider Clinical Trials	
	PLASTIC SURGEON CONSULTATION		If indicated	
	MEDICAL ONCOLOGIST		TREATMENT PLAN:<br>Teaching Visit<br>Follow-up visits between Chemotherapy Cycles	CHEMOTHERAPY:<br>Regimen:<br>Number of Weeks:<br><br>TARGETED TREATMENT:<br>Regimen:<br>Number of Weeks:
After cycle #6/8 or #4/6	BREAST SURGEON		Surgical Planning Visit<br>If indicated, order MRI<br>(after last cycle of chemo)	
At completion of chemo	RADIATION ONCOLOGIST			
	BREAST SURGEON			SURGERY<br>+/- Port Removal
2-3 wks. post-op	RADIATION ONCOLOGIST		Radiation Planning	RADIATION
2-3 wks. post-op	MEDICAL ONCOLOGIST		Discussion of additional treatments<br>If indicated	Hormone treatment<br>Targeted treatment<br>Clinical Trials
