# Supplementary material for: Outcomes of a Multidisciplinary Team in the Management of Patients with Early-Stage Breast Cancer Undergoing Neoadjuvant Chemotherapy at a Community Cancer Center
Source: Curr Oncol. 2023 May 8;30(5):4861–70. doi: 10.3390/curroncol30050366 (PMC10217230; doi:10.3390/curroncol30050366)
Supplement: Supplementary file 1 [file curroncol-30-00366-s001.zip › Supplementary tables.pdf]

Table S1: Indications for consideration of neoadjuvant systemic therapy:

Inoperable breast cancer:

- Inflammatory breast cancer
- Locally advanced breast cancer
  - N2, N3, T4
  - Fixed to chest wall or skin
  - Edema, Peau d'orange or ulceration

Operable breast cancer:

- Convert mastectomy to breast conservation surgery
- Allow less extensive surgery of the breast or axilla (Sentinel lymph node biopsy vs complete axillary dissection)
- Allow for less extensive radiation fields
- Allow time for genetic testing and planning
- Assess efficacy of treatment, prognosis (TN and HER2 positive) and tailor post-operative therapy
- Surgery needs to be delayed (pregnancy, recent extensive thrombosis, COVID-19)

For operable patients, consider based on the following tumor features:

- Clinical T2 or greater with any N
- Clinical T1N1
- All TN and HER2 positive except for T1a or T1b
- Carefully consider in ER positive patients

Caveats when considering neoadjuvant systemic therapy in operable patients:

- May still need mastectomy if large span of ductal carcinoma in-situ
- Caution if difficult to estimate extent of invasive disease
- Possibility of disease progression during systemic therapy (<5%)
- Risk of overtreatment
- ER positive may not need chemotherapy (Oncotype Dx testing)
- HER2 positive may be able to receive less intense regimen for e.g., T1N0 may receive weekly paclitaxel and trastuzumab

Table S2: Management after neoadjuvant chemotherapy

Characteristics	Overall	ER+ HER2+	ER+ HER2-	ER- HER2+	ER- HER2-
N (%)	94(100.0)	18 (19.1)	26 (27.7)	10 (10.6)	40 (42.6)
Downstaging of tumor – Yes	85 (91.4)	16 (88.9)	23 (92.0)	10 (100.0)	36 (90.0)
Downstaging of axilla – Yes	31 (33.0)	6 (33.3)	4 (15.4)	7 (70.0)	14 (35.0)
Surgery					
• Lumpectomy	53 (57.6)	8 (44.4)	14 (56.0)	5 (50.0)	27 (67.5)
• Mastectomy	39 (41.9)	10 (55.6)	11 (44.0)	5 (50.0)	13 (32.5)
• Bilateral Mastectomy	22 (23.7)	6 (33.3)	6 (24.0)	2 (20.0)	8 (20.0)
Axillary Management					
Sentinel Lymph Node Biopsy	59 (100)	9 (15.3)	10 (17.0)	9 (15.3)	31 (52.5)
Complete Axillary Node Dissection	34 (100)	9 (26.5)	15 (44.1)	1 (2.9)	9 (26.5)
Adjuvant Radiation					
• Yes	79 (84.0)	14 (77.8)	24 (92.3)	8 (80.0)	33 (82.5)
Areas of Radiation therapy					
Chest Wall	27 (28.7)	7 (38.9)	10 (38.5)	3 (30.0)	7 (17.5)
Breast	50 (53.2)	6 (33.3)	14 (53.8)	5 (50.0)	25 (62.5)
Internal Mammary	33 (35.1)	6 (33.3)	14 (53.8)	5 (50.0)	8 (20.0)
Supraclavicular	47 (49.5)	10 (55.6)	17 (65.4)	5 (50.0)	15 (37.5)
Axilla	46 (49.5)	10 (55.6)	16 (61.5)	6 (60.0)	14 (35.0)
Dosage of Radiation					
• Standard fractionation	42 (54.5)	7 (50.0)	16 (69.6)	6 (75.0)	13 (40.6)
• Hypofractionation	33 (42.9)	7 (50.0)	6 (26.1)	2 (25.0)	18 (56.3)
Adjuvant systemic therapy	66 (71.0)	18 (100.0)	24 (96.0)	9 (90.0)	15 (37.5)
• Chemotherapy	16 (17.0)	2 (11.1)	2 (7.7)	0 (0.0)	12 (30.0)
• Hormone therapy	39 (41.5)	14 (77.8)	24 (92.3)	1 (10.0)	0 (0.0)
• HER2 directed therapy	29 (30.9)	18 (100.0)	1 (3.8)	9 (90.0)	1 (2.5)

Abbreviations: ER: Estrogen receptor, HER2: Human Epidermal Growth Factor Receptor 2
